# Hypertrophic obstructive cardiomyopathy with multiple coronary arteries to right ventricular microfistulas: a case report and review of the literature 

**DOI:** 10.1186/s13256-016-1161-7

**Published:** 2017-01-31

**Authors:** Daulat Singh Meena, Chandra Bhan Meena, Javed Parvez

**Affiliations:** Department of Cardiology, Swai Man Singh Medical College, Jaipur, Rajasthan India

**Keywords:** Congenital coronary anomaly, HOCM, Angina in young, Coronary angiography

## Abstract

**Background:**

Coronary artery microfistulas are a rare anomaly; their association with hypertrophic cardiomyopathy is even rarer and can lead to serious cardiac complications owing to coronary steal phenomena such as angina pectoris, myocardial infarction, congestive heart failure, ventricular and supraventricular arrhythmias, syncope, and sudden death.

**Case presentation:**

A 32-year-old Indian woman presented to our institute with severe angina on exertion and multiple episodes of pre-syncope. Echocardiography revealed hypertrophic obstructive cardiomyopathy. Coronary angiography showed no significant atherosclerotic lesions; however, it revealed multiple microfistulas originated from all three major coronary arteries and draining into her right ventricle. This finding was confirmed by the rapid filling of the pulmonary artery after dye was injected into her left coronary artery during a cardiac catheterization study and by a significant oxygen step up of 15 % seen from her right atria to right ventricle during oximetry analysis. We treated our patient’s condition with medical therapy including metoprolol and nicorandil. She improved and angina grade had decreased from class III to class II on a follow-up visit 1 month after discharge.

**Conclusions:**

In this case report and literature review, we highlight an unusual but important association that can lead to symptomatic worsening of angina in young patients with hypertrophic cardiomyopathy owing to coronary steal phenomena.

**Electronic supplementary material:**

The online version of this article (doi:10.1186/s13256-016-1161-7) contains supplementary material, which is available to authorized users.

## Background

Coronary artery fistulas (CAFs) are a rare anomaly affecting the coronary arteries, with a prevalence of 0.13–0.2 %. They can cause serious cardiac complications such as angina pectoris, myocardial infarction, congestive heart failure, ventricular and supraventricular arrhythmias, syncope, and sudden death. Coronary artery microfistulas are even more rare and usually arise from either the right or left coronary system [1]. They most commonly present as angina on exertion but can also present with other symptoms that are similar to those of solitary CAFs. Their association with hypertrophic cardiomyopathy has been documented in eight previously published cases reporting altered symptomatology and atypical presentation.

To the best of our knowledge, this is first case report showing an association between hypertrophic obstructive cardiomyopathy (HOCM) and microfistulas arising from all three epicardial arteries in a patient presenting with angina and pre-syncope. In this case report, we aimed to document this important association that led to severe symptomatic worsening of angina on exertion owing to coronary steal phenomena.

## Case presentation

We report the case of a 32-year-old Indian woman admitted to our institute with angina on exertion. This symptom first appeared 2 years prior to her presentation, with gradual onset and progressive worsening from class I to class III associated with multiple episodes of pre-syncope. She had no history of diabetic mellitus, arterial hypertension, palpitation, or syncope and no family history of similar complaints or sudden death. On physical examination, our patient appeared generally well. Her blood pressure and pulse rate were 128/84 mmHg and 57 beats per minute, respectively.

### Investigations

**Fig. 1:**
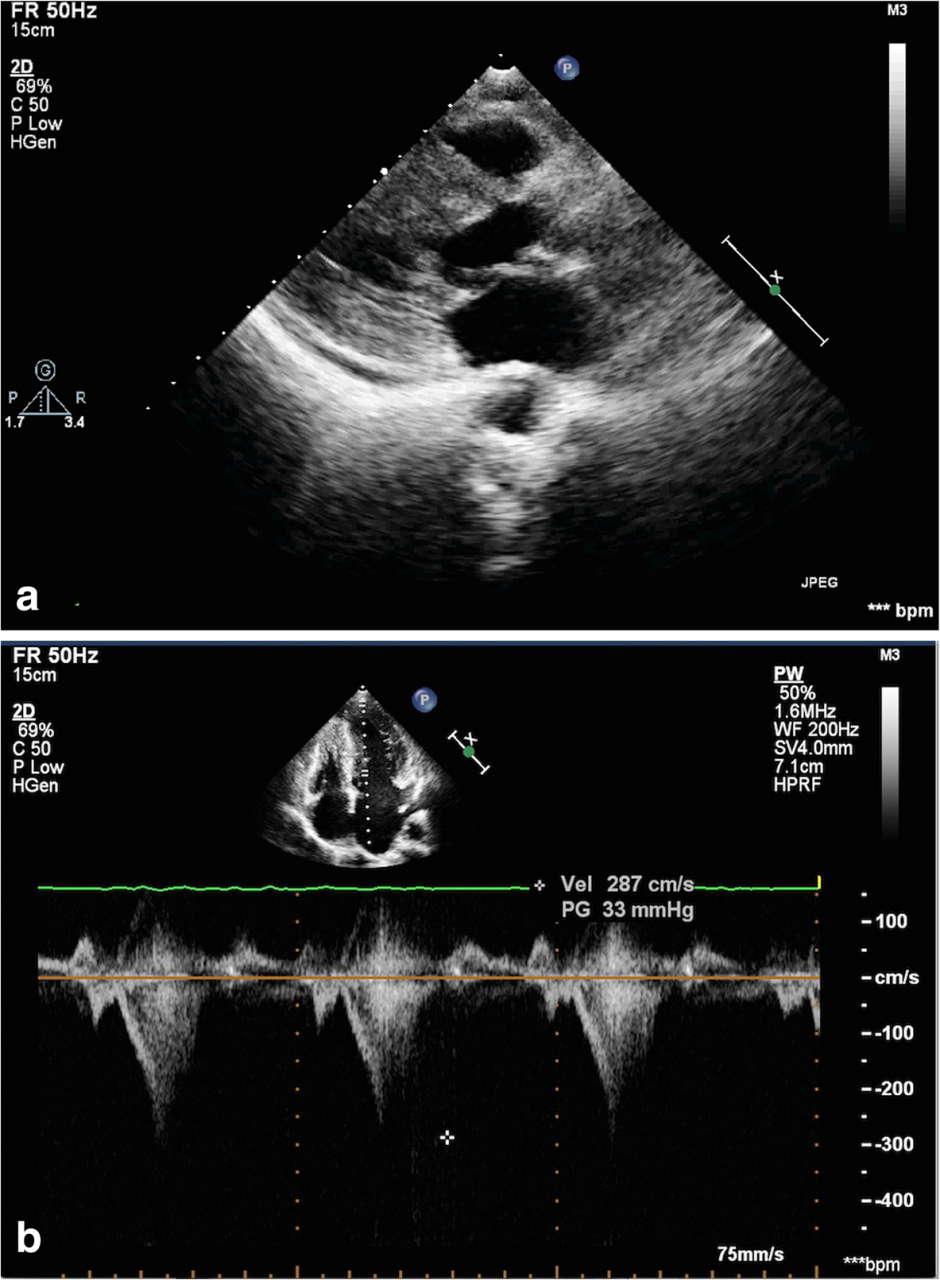
**a** Echocardiography revealed asymmetrical hypertrophic obstructive cardiomyopathy. **b** Pulsed wave Doppler suggested a resting sub-valvular obstruction with a pressure gradient of 33 mmHg

**Fig. 2:**
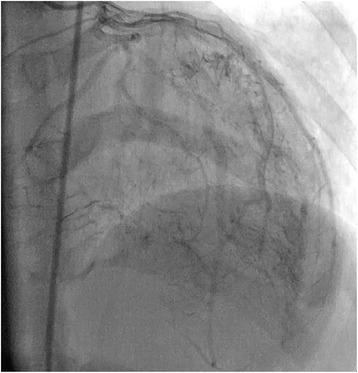
Multiple coronary artery microfistulas arising from the left anterior descending coronary artery and left circumflex artery

**Fig. 3:**
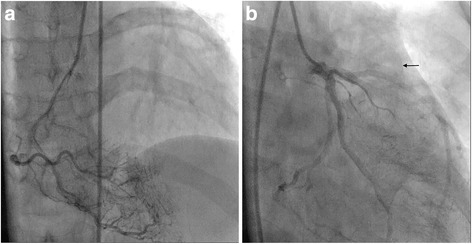
**a** Multiple coronary artery microfistulas arising from the right coronary artery to the right ventricle. **b** Left coronary artery microfistula draining to the right ventricle as shown by the filling of the artery (*black arrow*) from the left anterior descending coronary artery and left circumflex artery



Electrocardiography revealed a sinus rhythm and left ventricular hypertrophy by Sokolow–Lyon criteria, with secondary ST depression and biphasic T-waves in leads I, aVL, and V_4_–V_6_. A chest X-ray was normal. A Holter study was not suggestive of any significant arrhythmias or pause as a plausible cause of the pre-syncope. Her biochemical blood parameters were also within normal ranges. Echocardiography revealed HOCM (Fig. 1a) with systolic anterior movement. Pulsed wave Doppler showed a dagger-shaped spectral profile with a peak sub-aortic velocity of 287 cm/s and a maximum pressure gradient of 33 mmHg (Fig. 1b), with mild eccentric mitral regurgitation. After an initial evaluation, our patient underwent cardiac catheterization to exclude concomitant coronary artery disease and to rule out other coexisting congenital coronary anomalies_._ Coronary angiography showed no significant stenosis due to coronary artery disease. However, angiography revealed multiple coronary microfistulas originating from both her left coronary artery system (Fig. 2, Additional file 1) and her right coronary artery (Fig. 3a, Additional file 2). Our patient also underwent a cardiac catheterization study with oximetry analysis to confirm that the microfistulas originated from her left anterior descending coronary artery (LAD), left circumflex artery (LCX), and right coronary artery (RCA). The rapid filling of her pulmonary artery after dye was injected into her left coronary artery during the catheterization study demonstrated that the microfistulas were all draining into her right ventricle (RV) (Fig. 3b, Additional file 3). Further confirmation came from an oxygen step up of 15 % (from 65 % to 80 %) from her right atrium (RA) to her RV during oximetry analysis.

**Additional file 1:** Coronary angiogram




**Additional file 2:** Coronary angiogram




**Additional file 3:** Coronary angiogram


### Outcome and follow-up

Our patient was discharged 5 days after the completion of her clinical investigation on medical therapy including metoprolol and nicorandil. At a follow-up 1 month later, her angina grade had decreased from class III to class II.

## Discussion

Coronary artery microfistulas are congenital coronary artery anomalies that are associated with serious cardiac complications due to coronary steal phenomena. According to a recent Dutch survey, multiple coronary artery microfistulas are found in one quarter of all coronary artery fistulas detected by angiography; only one case has been reported in the age group of 20–50 years [2].

We present one of the first cases reported in the literature to date that shows an association between HOCM and multiple microfistulas arising from all three coronary arteries. None of the eight previously published case reports have shown microfistulas arising from all three coronary arteries (Table 1). The present study not only clearly identified dense microfistula arising from all three coronary arteries, but also confirming the receiving chamber via the significant oxygen step up from her RA to RV, along with rapid filling of her pulmonary artery by CAFs arising from her LAD and LCX. A case reported in Yildiz *et al.* [3] showed CAFs arising from the distal part of all three coronary arteries; however, the microfistulas reported in that case arose mostly from the distal septal branch of the LAD and not shown microfistulas arose from the distal LCX are separate from those arose from distil LAD.

**Table 1:** Comparative evaluation of all previously published case reports

Study	Age(Year)/Sex	Clinical presentation	Anatomy of fistulae
Present study	32/F	Angina on exertion, pre-syncope	LCX-RV, RCA-RV, LAD-RV
Yildiz *et al.* [3]	58/y	Stable angina	Distal septal branch of LAD-LV
			LCX-LV
			Distal RV branch of the RCA-LV
Alyan *et al.* [4]	63/M	Angina on exertion	LCA-LV
Hong *et al.* [5]	67/F	Dyspnea, angina on exertion	LCA-LV, RCA-LV
Dresios *et al.* [6]	82/F	ECG abnormality	LCA-LV
Caputo *et al.* [7]	6/M	Cardiac arrest	RCA-RV, RCA-PA
Monmeneu *et al. *[8]	NR/M	Angina on exertion	LCA-LV, RCA-LV
Delarche *et al. *[9]	NR	NR	Multiple coronary artery-LV fistulas
Kiyokawa *et al.* [10]	53/M	Angina	RCA-LV LCA-LV
	46/F		RCA-LV LCA-LV

*ECG* electrocardiography, *NR* not recorded, *M* male, *F* female, *LCX* left circumflex artery, *RV* right ventricle, *RCA* right coronary artery, *LAD* left anterior descending coronary artery, *LV* left ventricle, *LCA* left coronary artery, *PA* pulmonary artery

On angiography, CAFs are classified into two major types: solitary CAFs and coronary artery–ventricular multiple microfistulas. Solitary CAFs are defined as an abnormal connection between the coronary artery and any cardiac chamber or any part of the pulmonary or systemic circulation. They can be differentiated by identifying the origin, termination, and pathway. Coronary microfistulas are characterized as multiple fistulas of a small caliber that opacify the ventricular cavity; they are also known as generalized myocardial microfistulas.

In a Dutch registry, bilateral and multilateral fistulas were detected in 24 % and 4 % of the patients, respectively [2]. The pathogenesis of microfistula is obscure and usually attributed to a persistence of the embryonic myocardial sinusoids that originate from endothelial protrusions into intertrabecular spaces; fetal regression of these protrusions results in the formation of the Thebesian vessels. Thus interference with the developmental of embryonic myocardial sinusoids produces multiple coronary microfistulas [4–10]. Previous case reports have shown the RCA as the most frequent site of origin [6], whereas more recent studies suggest that the left coronary system is the more frequent site of origin, most commonly draining to the RV [7] and pulmonary artery.

Patients usually have an atypical presentation that depends on many factors, such as age of patient, amount of shunting, degree of coronary steal leading to cardiac ischemia, and resistance of recipient chamber. The majority of these patients are asymptomatic; in symptomatic patients, chest pain is the most common presenting symptom. In a study by Durán *et al.*, 57 % of 51 patients with CAFs with no underlying coronary artery disease had angina pectoris due to coronary steal [11]. Other symptoms include rupture or thrombosis of fistula, coronary arterial aneurysm, pulmonary artery hypertension, and congestive heart failure [6].

Coronary angiography remains the gold standard and helps to define the artery of origin, the recipient chamber, and the site of communication. Combined two-dimensional and pulsed Doppler echocardiography is useful only in cases of solitary CAF, in which they demonstrate a dilated coronary artery and turbulent flow in the fistula, and identify the recipient chamber [6]. Magnetic resonance imaging and multi-detector computed tomography are alternative noninvasive methods to evaluate the anatomy of both solitary macro- and microfistulas.

There are limited data available regarding the management of CAFs. Management is largely determined on the basis of the anatomical type of fistula, the amount of shunting, and any secondary complications caused by progressive enlargement of the fistula, such as bacterial endocarditis, thromboembolism, and pulmonary artery hypertension. All patients with hemodynamically significant solitary CAFs should undergo closure if they become symptomatic or develop complications. However, the amount of flow that is considered hemodynamically significant is still largely unknown, although a pulmonary to systemic flow ratio >1.5 is usually considered significant [12]. Previously, the only form of treatment available for coronary fistula after their identification was surgical ligation. However, subsequent to the first non-operative occlusion of a large coronary artery-to-bronchial anastomosis described by Reidy *et al.* (using a detachable balloon), catheter-based interventional techniques have become popular [13]. Over time, a variety of percutaneous techniques has emerged. Armsby *et al.* published a case series of 33 patients in whom transcatheter closure included coils in 28 patients, umbrella devices in six patients, and a Grifka vascular occlusion device in one patient; the different techniques showed similar early effectiveness, morbidity, and mortality compared to surgical ligation [14]. Currently, many catheter-based interventional methods are used. Coils are used primarily in smaller CAFs, because they offer the advantages of smaller sheath and catheter delivery sizes as well as a lower cost; double umbrella devices are used in larger fistulae because they allow more precise positioning, especially in cases where the coronary branches are close to the occlusion site [14, 15].

There are no data available on the management of patients with HOCM with coronary microfistula owing to the rarity of this disease. Treatment of isolated HOCM with coronary microfistula is essentially by medical management. We discharged our patient on medical therapy with metoprolol and nicorandil. She showed significant symptomatic improvement and an angina grade decrease from class III to class II on a follow-up visit 1 month after discharge.

## Conclusions

Our case contributes to the growing evidence regarding the rare association of microfistulas from multiple coronary arteries to the RV with HOCM. Understanding the pathophysiological implications of this association on clinical presentation and symptomatic progression will enhance the management of theses unusual cases.

## Abbreviations

CAF: Coronary artery fistula
HOCM: Hypertrophic obstructive cardiomyopathy
LAD: Left anterior descending coronary artery
LCA: Left coronary artery
LCX: Left circumflex artery
LV: Left ventricle
RA: Right atrium
RCA: Right coronary artery
RV: Right ventricle
